# The Plasticity of Brain Gray Matter and White Matter following Lower Limb Amputation

**DOI:** 10.1155/2015/823185

**Published:** 2015-10-25

**Authors:** Guangyao Jiang, Xuntao Yin, Chuanming Li, Lei Li, Lu Zhao, Alan C. Evans, Tianzi Jiang, Jixiang Wu, Jian Wang

**Affiliations:** ^1^Department of Radiology, Southwest Hospital, Third Military Medical University, Chongqing 400038, China; ^2^McConnell Brain Imaging Centre, Montreal Neurological Institute, McGill University, Montreal, QC, Canada H3A 2B4; ^3^Department of Rehabilitation, Southwest Hospital, Third Military Medical University, Chongqing 400038, China; ^4^National Laboratory of Pattern Recognition, Institute of Automation, Chinese Academy of Sciences, Beijing 100190, China

## Abstract

Accumulating evidence has indicated that amputation induces functional reorganization in the sensory and motor cortices. However, the extent of structural changes after lower limb amputation in patients without phantom pain remains uncertain. We studied 17 adult patients with right lower limb amputation and 18 healthy control subjects using T1-weighted magnetic resonance imaging and diffusion tensor imaging. Cortical thickness and fractional anisotropy (FA) of white matter (WM) were investigated. In amputees, a thinning trend was seen in the left premotor cortex (PMC). Smaller clusters were also noted in the visual-to-motor regions. In addition, the amputees also exhibited a decreased FA in the right superior corona radiata and WM regions underlying the right temporal lobe and left PMC. Fiber tractography from these WM regions showed microstructural changes in the commissural fibers connecting the bilateral premotor cortices, compatible with the hypothesis that amputation can lead to a change in interhemispheric interactions. Finally, the lower limb amputees also displayed significant FA reduction in the right inferior frontooccipital fasciculus, which is negatively correlated with the time since amputation. In conclusion, our findings indicate that the amputation of lower limb could induce changes in the cortical representation of the missing limb and the underlying WM connections.

## 1. Introduction

Human brain plasticity or neuroplasticity refers to the capacity of the nervous system to modify the organization of the brain structure and function in response to experience. It is an intrinsic property of the nervous system retained throughout a lifespan [1]. Previous studies suggested that both short-term [2, 3] and long-term training [4–6] can modulate brain structural changes involved with both the gray matter (GM) and white matter (WM). The candidate mechanisms for these changes are multifaceted and likely include gliogenesis, synaptogenesis, and vascularization in GM, as well as myelination and axonal sprouting in WM [7].

In addition to normal training or experience, a growing body of evidence has accumulated supporting injury-induced functional or structural plasticity at different levels in the adult central nervous system [8–10]. Previous studies suggest that, at least in primates, plasticity in the cortical representation can occur rapidly as a consequence of peripheral lesions or sensory deprivation [11, 12]. As a drastic limb injury, amputation in humans has been reported to lead to extensive reorganization, most prominently in the primary somatosensory and motor areas, which was suggested to correlate with phantom limb pain (PLP) [13–16]. Despite extensive neurobiological research, the underlying nature of such phenomena remains elusive. While some authors have argued that cortical reorganization following amputation is triggered by the loss of sensory input [16, 17], others have proposed that the mechanisms should be attributed to the persistent experience of pain [18]. These discrepancies in the literature raise the fundamental question of whether brain reorganization occurs in amputees without PLP. On the other hand, it also should not be overlooked that the short- and long-term effects of amputation on the brain may be varied, as PLP is usually more common in the initial stage after amputation [19].

Amputees have been found to have structural differences in both GM and WM. One study using automated voxel-based morphometric analysis found that subjects with limb amputation exhibited a GM decrease in the thalamus, which was unrelated to PLP [20]. However, this investigation did not distinguish between upper and lower limb amputation. In addition, reduced GM volume in the primary motor [21] or sensory [18] cortices was also observed in patients with amputation or spinal cord injury. In contrast to voxel-based morphometry, the measurement of cortical thickness provides a more direct and meaningful index. Preißler and colleagues [22] found that cortical thickness in upper limb amputees was reduced in the motor cortex but increased in the temporal and parietal lobes. Although GM reorganization was initially the focus of many brain imaging studies, WM changes after limb amputation are increasingly being investigated using neuroimaging techniques, especially diffusion tensor imaging (DTI), which provides information about WM tracts and their organization based on water diffusion. Fractional anisotropy (FA) is the most often used DTI index of WM integrity, and reduced FA in amputees has been reported in the corpus callosum (CC) and corticospinal tract [23]. Although these studies have been carried out to determine the effects of missing limbs on brain reorganization, little is known about the associations between GM and WM changes after amputation.

The purpose of this study was to examine the long-term patterns of brain reorganization following limb amputation. To systematically characterize brain reorganization, we first used a combined tract-based spatial statistics (TBSS) and tractography analysis, which enables a precise characterization of both whole-brain WM and specific anatomical fiber tracts, to assess the microstructural changes in patients with unilateral amputation in the lower limb. We then performed surface-based morphometry across the whole brain GM and regions of interest (ROI) focusing on the sensorimotor cortices. Finally, the relationships between GM and WM changes in amputees were investigated.

## 2. Materials and Methods

### 2.1. Subjects

Seventeen adult patients (13 males and 4 females) with right lower limb amputation were recruited from the Prosthetic and Orthotic Clinics at the Department of Rehabilitation, Southwest Hospital in Chongqing. All the patients had been fitted with prostheses. Twelve were amputations following traumatic injury and five were due to tumors (2 being melanoma and 3 being osteosarcoma). Ten amputations occurred at the transtibial and seven at transfemoral levels. Exclusion criteria were the following: (1) age at amputation of less than 18 years or more than 60 years; (2) amputation at another part of the body; (3) presence of major systemic disease (e.g., diabetes mellitus, cardiovascular diseases, and inflammation), psychiatric or neurological illnesses; (4) duration between amputation and magnetic resonance imaging (MRI) scanning of less than 6 months; (5) presence of PLP or stump pain assessed by the five-category verbal rating scale [24].

Eighteen age- and sex-matched healthy controls without neurological or psychiatric diseases and with normal brain MRI were recruited from the local community. All the participants were dominantly right-handed as determined by the Edinburgh Handedness Inventory [25] and had a score of 27 or higher on the Chinese version of the Mini-Mental Status Examination (MMSE) [26]. The study was approved by the Medical Research Ethics Committee of Southwest Hospital, and written informed consent was obtained from all participants.

### 2.2. Imaging Data Acquisition

All of the participants were scanned using a 3.0 Tesla imager (Tim Trio, Siemens, Erlangen, Germany) with a 12-channel head coil. DTI data were acquired using a single-shot twice-refocused spin-echo diffusion echo planar imaging sequence (repetition time = 10,000 ms, echo time = 92 ms, 64 nonlinear diffusion directions with *b* = 1000 s/mm^2^, and an additional volume with *b* = 0 s/mm^2^, matrix = 128 × 124, field of view = 256 × 248, and 2 mm slice thickness without gap). From each participant 75 axial slices were acquired and the diffusion sequence was repeated twice to increase the signal-to-noise ratio. T1-weighted three-dimensional magnetization-prepared rapid gradient echo images were then collected using the following parameters: repetition time = 1,900 ms, echo time = 2.52 ms, inversion time = 900 ms, flip angle = 9°, matrix = 256 × 256, thickness = 1.0 mm, and 176 slices with voxel size = 1 × 1 × 1 mm^3^.

### 2.3. DTI Data Analysis

The DTI data were preprocessed using the FMRIB Software Library (University of Oxford, UK). First, the diffusion data were corrected for eddy currents and head motion, and the two acquisitions were averaged. The averaged images were masked to remove skull and nonbrain tissue using the FSL Brain Extraction Tool [27]. Then, the diffusion parametric images were calculated using the diffusion tensor analysis toolkit [28].

Data were then prepared for statistical analysis using TBSS [27]. First, FA images for all subjects were nonlinearly aligned to a study-specific minimal-deformation target (MDT) brain and resampled to an isotropic 1 mm resolution. The MDT brain was selected as the brain image that minimizes the deformation from other brain images in the group through warping all FA images in the group to each other [29, 30]. Next, the mean FA image was created and thinned to create a mean FA skeleton that represents the centers of all fiber tracts. The FA threshold of 0.2 was chosen to restrict the skeleton to WM tracts. Each subject's aligned FA data were then projected onto this skeleton.

### 2.4. Probabilistic Diffusion Tractography (PDT)

Clusters showing group differences in the TBSS analysis were used as seed masks for multifiber probabilistic tractography [31] in each subject's native space. The steps have been described in detail in our previous articles [32, 33]. For each participant, PDT was run from each voxel in the seed mask to the whole brain using default parameters. The warp fields of nonlinear registration and the inverse versions were used for the translation between the original space and the standard space. For the elimination of spurious connections, the individual tracts in standard space obtained by PDT were arbitrarily thresholded to include only voxels through which at least 25% (1,250) of samples had passed. Each subject's tracts were then binarized and summed to produce group probability maps for each pathway. The group probability maps were also thresholded at 25% (at least 9 of the 35 subjects) to generate the masks for each fiber pathway. The WM labels atlas [34] and tractography atlas [35] implemented in FSL were used for the structural identification. Individual mean FA values of each pathway were then extracted from the standardized whole-brain DTI images.

### 2.5. Cortical Thickness Analysis

All the structural T1 images were analyzed using FreeSurfer (version 5.3.0, https://surfer.nmr.mgh.harvard.edu/) to create anatomical surface models. The automated processing stream mainly included removal of nonbrain tissue [36], Talairach transformation, segmentation of gray/white matter tissue [37], intensity normalization, topological correction of the cortical surface [38], and surface deformation to optimally place the tissue borders [39]. The tissue boundaries were reviewed and manually edited for technical accuracy. Cortical thickness was calculated as the shortest distance between the GM and WM surfaces at each vertex across the cortical mantle. Moreover, the GM volume in each hemisphere and total intracranial volume (TIV) was also calculated from the FreeSurfer processing stream.

Finally, using the Brodmann Areas (BA) atlas in FreeSurfer (https://surfer.nmr.mgh.harvard.edu/fswiki/BrodmannAreaMaps), we measured the individual mean cortical thickness values in the sensorimotor regions, including the bilateral BA 1, 2, 3a, 3b, 4a, 4p, and 6. In order to avoid the overlap among these labels, they were all thresholded at 80% probability.

### 2.6. Statistical Analyses

Group differences in age, years of education, and neuropsychological scores were examined using independent samples *t*-tests. Sex data were analyzed with a chi-square test. Differences in FA between the amputees and controls were determined using the FSL “randomize” tool, which is specifically designed for permutation testing with nonparametric values. Age and sex were used as the covariates. Clusters were reported reaching a significance level of *P* < 0.05, corrected for multiple comparisons across image using the null distribution of the maximum cluster mass (*t* > 3) [32]. Cluster mass is the sum of all statistic values within the cluster and has been reported to be more sensitive than cluster size [40].

Whole-brain vertex-wise group comparisons for cortical thickness were performed on a standardized surface [41] and the data were smoothed using a full-width/half-maximum Gaussian kernel of 10 mm on the surface. Regional differences between amputees and controls were assessed using a vertex-by-vertex general linear model controlling for the potential confounding effects of age, sex, and TIV. The statistical analyses were performed with the SurfStat toolbox based on Random Field Theory (RFT) [42]. Clusters were first reported reaching a significant level of RFT-corrected *P* < 0.05, and then those reaching a looser significance level of uncorrected *P* < 0.005 were also indicated.

Analyses of covariance (ANCOVA) adjusting for age and sex were used to explore the group differences in the mean FA value for each of the fiber tracts generated by PDT and in the mean cortical thickness for each of the selected sensorimotor regions in both hemispheres. Finally, the relationships between the WM and GM changes were investigated using partial correlation analyses (adjusted for age and sex). A false discovery rate (FDR) corrected threshold of 0.05 was considered as significant for these analyses.

## 3. Results

### 3.1. Demographics and Clinical Measures

Demographic and relevant clinical information is listed in Table 1. There were no significant differences in sex ratio, age, education, and MMSE scores between the amputees and controls.

### 3.2. WM Differences Revealed by TBSS and PDT

**Figure 1:**
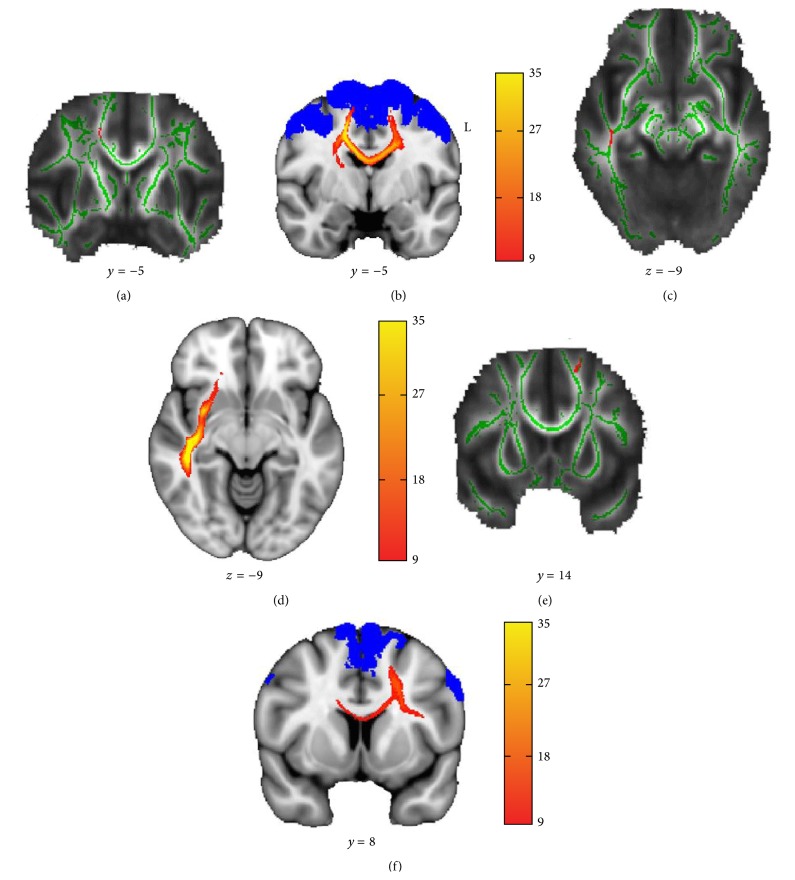
Results of TBSS analysis of FA maps (a, c, and e) and group probability maps (b, d, and f) from the corresponding regions. The mean white matter FA skeleton is shown in green. The blue mask indicates the PMC obtained from the Jülich histological atlas. The group probability maps were thresholded at 25% (at least 9 persons from the 35 subjects) and the color bar indicates the number of participants in whom the generating fiber pathways pass through that voxel.

Compared with controls, the amputees showed a decreased FA in the right superior corona radiata and WM regions underlying the right temporal lobe and left premotor cortex (PMC) (Figures 1(a), 1(c), and 1(e); Table 2). No FA increase was found in amputees relative to controls.

PDT from the above clusters revealed that the contributing WM tracts were the commissural fibers connecting the bilateral premotor cortices and the association fibers that exactly overlapped with the inferior frontooccipital fasciculus (IFOF) (Figures 1(b) and 1(d)). The cluster underlying the left PMC also generated local premotor and transcallosal paths (Figure 1(f)).

The results of ANCOVA demonstrated that the mean FA values extracted from the thresholded group probability maps in amputees were all significantly reduced (*P* < 0.05, FDR correction for multiple comparisons) in all the fiber tracts (Table 3).

### 3.3. Cortical Thickness Differences

**Figure 2:**
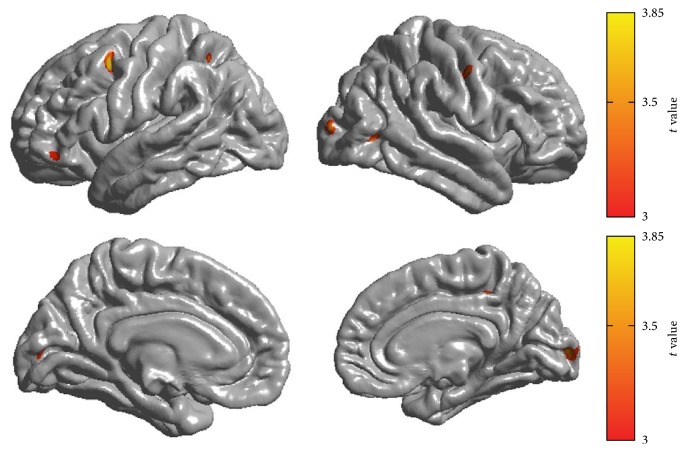
Regional cortical thinning in amputees compared with the controls. *P* < 0.005 (*t* > 3), uncorrected.

The GM volume (controls versus amputees: left, 0.25 ± 0.02 versus 0.24 ± 0.03 L, *P* = 0.13; right, 0.25 ± 0.02 versus 0.24 ± 0.03 L, *P* = 0.17) and TIV (1.56 ± 0.15 versus 1.51 ± 0.14, *P* = 0.17) of amputees did not differ significantly from those of controls. The amputees showed a thinning trend (*P* < 0.005, uncorrected) in different cerebral lobules, with the largest one in the left PMC. Smaller clusters of cortical thinning were also noted in the bilateral occipital lobes, the right temporooccipital junction, precentral gyrus, precuneus lobe, the left inferior parietal lobule, and frontal orbital cortex (Figure 2; Table 4). However, no clusters survived after RFT correction for multiple comparisons. We did not find any clusters exhibiting thickness increase in the amputees compared with the control group (*P* < 0.005, uncorrected).

The results of ANCOVA for the ROI confirmed that the cortical thickness was only significantly decreased in the left premotor area (BA 6) in the amputees relative to the controls (2.73 ± 0.14 versus 2.84 ± 0.12; *P* = 0.02). The difference remained significant (*P* = 0.03) even when we added TIV as an extra covariate (Table 5).

### 3.4. Associations between WM and GM Changes in Amputees

No significant associations were found between the cortical thickness in the affected regions (as shown in Table 4) and the DTI parameters of the fiber tracts generated from the PDT in the amputees. However, partial correlation analyses revealed that the FA value of the IFOF (as shown in Figure 1(d)) was negatively correlated to the time since amputation (*r* = −0.55, *P* = 0.03).

## 4. Discussion

In the present study, we explored brain structural reorganization in lower limb amputees without PLP. Cortical thickness and FA values were used as measures to evaluate the GM and WM microstructural changes across the whole brain compared with normal controls. As a consequence, we found that patients with amputation at the right lower limb exhibited cortical thinning in the left premotor area and the right visual-to-motor regions. Additionally, the integrity of the fiber tracts connecting the bilateral PMC and those underlying the right visual-to-motor regions was also significantly reduced in the patients.

Our study demonstrates that cortical reorganization occurs in lower limb amputees, even in the absence of PLP. We observed a thinning trend in different cerebral lobules, especially in the PMC contralateral to the affected side. The PMC encompasses the anterior lip of the precentral gyrus, the posterior portion of the middle frontal gyrus, and the superior frontal gyrus on the superolateral surface of the brain, corresponding to part of BA 6 [43]. The time-specific studies of the PMC and primary motor cortex reflect the distinct roles of the two areas: the PMC is involved in movement selection, whereas the latter is involved in movement execution [44, 45]. The activity of PMC neurons is also responsible for the specification of movement parameters such as amplitude, direction, and speed of movement [43]. Additionally, the PMC also seems to be involved in the control of eye movements and eye-related neural activity or in specific tasks that require eye-limb coordination [46, 47]. As amputation in the lower limb will lead to a lack of movement selection and disorders of movement parameters and coordination, it can be inferred that the GM loss in the PMC following amputation is possibly attributed to long-term use-dependent blockage.

Reduced GM volume in the left primary motor cortex had also been reported in patients with right upper limb amputation [22] but was not found in the current lower limb amputees. One possible reason for the discrepancy could be the sample heterogeneity between studies. As the upper limb representation is much bigger than the lower limb in the brain [48], the morphological changes due to functional nonuse could be less significant for the patients with lower limb amputation. In line with our reports, one previous study, in which 19 of the 28 patients were amputated at the lower limb, also did not find alterations in the primary motor cortex [20]. In addition, reorganization in the primary somatosensory and motor areas after amputation has been suggested to correlate with PLP [15, 16]. In our study, the amputees with PLP were not included. Therefore, our findings would provide an update on the distinctive patterns of brain plasticity in lower limb amputees without PLP.

In this study, TBSS allowed us to obtain subcortical WM changes across the whole brain in amputees. The PDT approach was used to reconstruct the tracts from the WM skeleton regions characterized by FA decrease in lower limb amputees. This allowed the investigation of abnormal structural connectivity. Our TBSS analysis revealed that right lower limb amputees displayed significant FA reduction in the right superior corona radiata and WM underlying the left PMC. Further fiber tracking generated the transcallosal paths linking the homologous PMC of the bilateral hemispheres. These findings are very consistent with one pioneering DTI study, which reported the reduced integrity in the body of the CC in amputees [23]. It is known that unilateral movement requires sequential processing in bihemispheric motor areas. Using transcranial magnetic stimulation, previous studies found that the PMC modulates the activity of contralateral motor areas during the preparatory period of a voluntary movement with the ipsilateral limb [49, 50]. Such modulation is mediated by interhemispheric inhibition through fibers within the CC [51] and enables healthy adults to perform complex motor tasks without the activation of contralateral muscles [52]. Therefore, the FA reduction within the CC connecting the bilateral PMC may reflect adaptive WM modification following the changes of movement patterns, as the transcallosal inhibition function is disused in unilateral lower limb amputees.

Beyond the left PMC, smaller clusters of cortical thinning in amputees were also noted, mainly in the brain regions constituting visual-to-motor networks, including the bilateral visual cortices, the right temporooccipital junction, left inferior parietal lobule, and orbital frontal cortex. Functional MRI has found that human parietal and temporooccipital cortices constitute the core nodes for cross-modal vision-action representations [38]. Meanwhile, the inferior parietal lobule, particularly in the left hemisphere, contributes to motor attention and is activated in neuroimaging experiments when subjects prepare movements or switch intended movements [53]. Contralateral atrophy in the parietal lobe has also been reported in upper limb amputees [54]. Visual-motor transformation also engages the orbital frontal cortex, which becomes active during response preparation and execution [55]. Further functional/structural connectivity studies confirm that the PMC integrates visual and somatosensory information from the intraparietal area to allow effective exchange and elaboration of information [56]. The connections within the neural networks are plastic and are modified in response to injuries [57, 58], training, and treatments [59]. Previous imaging studies demonstrated that stimulation of afferent input could result in functional reorganization and a corresponding structural expansion of the cortical and subcortical areas [2, 60]. Accordingly, the loss of afferent input following limb amputation should cause “negative” structural alterations with a decrease in GM.

Lower limb amputees also display significant FA reduction in the right IFOF, which is negatively correlated with the time span after amputation. The IFOF connects the inferior frontal lobe to the posterior temporal-occipital regions and provides the main anatomical connections for the ventral (bottom-up) attention system [61], which is specialized for the detection of behaviorally relevant stimuli [62]. Reduction of WM integrity in the IFOF has been reported to be associated with deficits in executive function in patients with chronic trauma [63]. Furthermore, our previous DTI study showed that the right hemispheric IFOF confers an advantage for the executive function of attention [33], which is in line with the well-described rightward dominance of visuospatial processing [64]. Interestingly, the amputees presented time-related microstructural abnormalities of the IFOF in the right rather than the left hemisphere, indicating the degenerative function of visuospatial processing following amputation [65]. Future studies including neuropsychological assessments should be used to investigate the underlying explanations for the associations between brain WM plasticity and visuospatial function in amputees.

The negative findings of GM increase are supported by one MRI study [20] but are incongruent with another [22]. The differences might be due to the status of PLP, prosthesis use, amputation sites, or time span after amputation. Using a smaller sample size, Preißler et al. [22] found that upper limb amputees with slight PLP showed GM increase in regions of the visual stream. They initially hypothesized that it might be a compensatory effect for the lack of sensorimotor feedback and could serve as a protection mechanism against high PLP development [22]. However, in their following study using the same patients, a negative association between prosthesis use and cortical volume in the posterior parietal and occipital lobes, which greatly overlap with the regions with GM loss in our findings, was reported [66]. As prosthesis use has been shown to have a beneficial influence on the prevention of cortical reorganization and PLP [67, 68], and patients rely less often on bottom-up or stimulus-driven control with increasing prosthesis use [66]; we could speculate that the cortical thinning and FA reductions in the ventral visual stream also reflect adaptive brain plastic changes along with the transformation of human abilities and might be beneficial for the prevention of PLP.

Although these findings are robust, some limitations of the present study need to be addressed. First, the relatively small sample size in this study may mask subtle differences between groups, especially in the vertex-based cortical thickness analysis across the whole brain. Therefore, the uncorrected results were reported to minimize type II errors. Second, we only included patients with amputation at the right side. Left lower limb amputation might result in different morphological and functional changes, especially with respect to the contralateral PMC and the structures in the visual stream. It will be of interest to determine whether the individuals with amputation at the left side will demonstrate the analogous changes at the homologous regions of the other hemisphere. Finally, our explanation of reduced interhemispheric inhibition in amputees is just speculative. Future studies should be performed to confirm the interhemispheric interactions using noninvasive transcranial current or magnetic stimulation.

## 5. Conclusion

In this study, we combined high-resolution brain structural MRI and DTI to investigate the existence and extent of cortical and WM plasticity in subjects with right lower limb amputation. In summary, we found specific motor and somatosensory plastic changes in amputees without PLP and provided an update on the plasticity of the human brain involving both GM and underlying WM after limb injury.

## Figures and Tables

**Table 1:** Demographic characteristics of the participants.

	Patients	HC	*P* value
Age (years)	37.5 ± 13.5 (18–60)	37.0 ± 12.7 (19–60)	0.91
Male : female	13 : 4	13 : 5	0.54
Education level (years)	9.5 ± 2.7 (6–15)	9.6 ± 3.3 (5–16)	0.94
Age at amputation (years)	32.9 ± 12.6 (18–59)	—	—
Time since amputation (months)	71.4 ± 102.4 (7–336)	—	—
MMSE score	28.0 ± 1.4 (27–30)	28.4 ± 1.2 (27–30)	0.37

The data were presented as mean ± SD (range). HC, healthy controls; MMSE, Mini-Mental Status Examination.

**Table 2:** Regions showing significant FA reduction in the amputees.

Region	Cluster index	Hemisphere	MNI coordinates			Voxels	*P* value
			*x*	*y*	*z*		
Superior corona radiata	3	R	17	−6	38	105	0.03
Temporal WM	2	R	43	−24	−13	95	0.03
WM underlying PMC	1	L	−15	14	50	76	0.04

The output was thresholded at cluster level (*t* > 3) and corrected for multiple comparisons using the null distribution of the maximum (across image) cluster size (*P* < 0.05). MNI, Montreal Neurological Institute; PMC, premotor cortex; WM, white matter.

**Table 3:** The differences of FA values in fiber tracts generated from tractography.

Region	Cluster index	FA value		*P* value
		Controls	Patients	
WM connecting bilateral PMC	3	0.48 ± 0.02	0.44 ± 0.04	0.009
Right IFOF	2	0.47 ± 0.03	0.45 ± 0.02	0.009
WM underlying left PMC	1	0.36 ± 0.02	0.33 ± 0.02	0.0003

The *P* value was adjusted for multiple comparisons. IFOF, inferior frontooccipital fasciculus; PMC, premotor cortex; WM, white matter.

**Table 4:** Regions showing significant differences of cortical thickness across the whole brain.

Region	H	BA	Coordinates			Mean thickness		*P* value	Peak *T* score	Vertex number
			*x*	*y*	*z*	Patients	Controls			
PMC	L	6	−41	6	54	2.44 ± 0.40	2.81 ± 0.27	0.001	3.84	169
V1	R	17	12	−96	4	1.68 ± 0.16	1.89 ± 0.21	0.001	3.64	167
TOJ	R	37	43	−65	2	2.20 ± 0.15	2.45 ± 0.21	0.001	3.49	127
preCG	R	4	52	−8	43	2.62 ± 0.24	2.83 ± 0.16	0.001	3.55	108
V2/V3	R	18	30	−95	8	1.88 ± 0.21	2.14 ± 0.19	0.001	3.52	104
Precuneus	R	N.A.	16	−37	46	2.01 ± 0.17	2.22 ± 0.17	0.001	3.38	94
V1	L	17	−10	−88	6	1.42 ± 0.11	1.62 ± 0.19	0.003	3.40	89
IPL	L	7	−29	−71	40	2.12 ± 0.16	2.33 ± 0.16	0.001	3.76	86
OFC	L	47	−46	42	−10	1.94 ± 0.21	2.14 ± 0.21	0.002	3.17	59

The results were reported at *P* < 0.005 (uncorrected) and vertex number >50. BA, Brodmann Area; H, hemisphere; IPL, inferior parietal lobule; N.A., not available; OFC, orbital frontal cortex; PMC, premotor cortex; preCG, precentral gyrus; TOJ, temporooccipital junction; V1, primary visual cortex; V2/V3, extrastriate visual areas 2/3.

**Table 5:** The differences of mean cortical thickness in the sensorimotor cortices between the amputees and normal controls.

Region	Hemisphere	Mean cortical thickness		*P* value^a^	*P* value^b^
		Controls	Patients		
BA 1	L	2.27 ± 0.22	2.16 ± 0.21	0.14	0.19
	R	2.25 ± 0.26	2.21 ± 0.21	0.65	0.73
BA 2	L	2.17 ± 0.19	2.08 ± 0.18	0.17	0.24
	R	2.11 ± 0.15	2.05 ± 0.17	0.27	0.46
BA 3a	L	1.67 ± 0.09	1.66 ± 0.10	0.79	0.86
	R	1.67 ± 0.10	1.65 ± 0.09	0.61	0.71
BA 3b	L	1.53 ± 0.09	1.49 ± 0.09	0.18	0.15
	R	1.54 ± 0.11	1.52 ± 0.12	0.6	0.61
BA 4a	L	2.74 ± 0.15	2.63 ± 0.21	0.06	0.09
	R	2.85 ± 0.19	2.77 ± 0.17	0.21	0.22
BA 4b	L	2.41 ± 0.17	2.31 ± 0.20	0.13	0.15
	R	2.39 ± 0.15	2.35 ± 0.20	0.53	0.40
BA 6	L	2.84 ± 0.12	2.73 ± 0.14	**0.02**	**0.03**
	R	2.83 ± 0.12	2.76 ± 0.18	0.20	0.23

^a^Adjusted for age and sex; ^b^adjusted for age, sex, and total intracranial volume. BA, Brodmann Area; L, left; R, right. Bold indicates *P* < 0.05 (FDR correction for multiple comparisons).
